# Determining parental origin of embryo aneuploidy: analysis of genetic error observed in 305 embryos derived from anonymous donor oocyte IVF cycles

**DOI:** 10.1186/s13039-014-0068-5

**Published:** 2014-10-25

**Authors:** E Scott Sills, Xiang Li, Jane L Frederick, Charlotte D Khoury, Daniel A Potter

**Affiliations:** HRC Fertility—Orange County, 500 Superior Ave., Suite 210, Newport Beach, CA 92663 USA; Division of Applied Biotechnology, Faculty of Science & Technology, University of Westminster, London, UK; Division of Analytics & Quantitative Research, Rosenblatt Securities Inc, New York, NY USA

## Abstract

**Background:**

Since oocyte donors are typically young and believed to be a source of highly competent gametes, donor oocyte IVF is considered to be an effective treatment for diminished ovarian reserve. However, the aneuploidy rate for embryos originating from anonymously donated oocytes remains incompletely characterized. Here, comprehensive chromosomal screening results were reviewed from embryos obtained from anonymous donor-egg IVF cycles to determine both the aneuploidy rate and parental source of the genetic error. To measure this, preimplantation genetic screening (PGS) data on embryos were retrospectively collated with parental DNA obtained before IVF for chromosome-specific assessments. This approach permitted mitotic and meiotic copy errors to be differentiated for each chromosome among all embryos tested, thus providing information on the parental source of embryo aneuploidy (*i.e.,* from the anonymous egg donor vs. sperm source).

**Results:**

305 embryos generated for 24 patients who began IVF treatment in 2013. For oocyte donors (*n* = 24), mean (±SD) age was 24.0 ± 2.7 years (range = 20-29). For embryos with full chromosomal reporting (*n* = 284), euploidy was present in only 133 (46.8%). Considering all embryo chromosomes, the average error rate was 18%. 133 of 151 observed embryo aneuploidies (88.1%) were attributable to an oocyte donor source. Among all aneuploid embryos (*n* = 151), chromosomal errors from both genetic parents (*i.e*., oocyte donor and sperm source) were present in 57%. The average correlation coefficient across all pairs of chromosomal abnormalities (*r* = 0.60) suggests that chromosomes tend to have multiple and simultaneous errors (complex aneuploidy) even when oocytes from young donors are used.

**Conclusion:**

These data show that even when young donors provide oocytes for IVF, the probability of embryo aneuploidy remains high. The oocyte donor appears to make an important contribution to embryo aneuploidy even when her age is <30 yrs. If these findings are confirmed with larger, prospective studies, the routine integration of PGS with donor oocyte IVF cycles to identify single euploid embryos for transfer should be considered.

## Background

Embryo aneuploidy is among the most important contributors to poor outcomes observed with in vitro fertilization (IVF). Numerous investigators have independently concluded that human embryos intrinsically contain substantial chromosomal error [1-3] and that this problem is more pronounced as maternal age increases. Accordingly, preimplantation testing of embryos obtained from IVF patients at age ≥35 can be helpful to improve efficiency of infertility treatment. Nevertheless, oocyte donation with IVF remains a commonly applied method of assisted reproduction for this group of older patients [4]. The therapeutic intervention of oocyte donation with IVF is believed to be so successful largely because oocyte quality is greatly improved when the donor’s age is low, thus yielding better pregnancy rates and reduced miscarriage risk.

But when oocytes are obtained from young, healthy, anonymous donors, how low does the chromosomal error rate actually go? This question has already been partially explored when a limited number of chromosomes were studied in embryos obtained from donor-egg IVF treatment, and the aneuploidy rate (even in that partial genomic assessment) was higher than expected [5]. From this earlier pioneering work, our study reviewed preimplantation genetic screening (PGS) data in the context of anonymous donor oocyte IVF. Using increased bandwidth to capture comprehensive chromosomal screening data on all 23 pairs of chromosomes, this investigation aimed to answer two unresolved issues: 1) What is the actual incidence of genetic abnormality in embryos produced from anonymously donated oocytes, and 2) Did the embryo ploidy error originate from the sperm of the recipient’s partner, or the oocyte of the anonymous donor?

## Results

**Figure 1:**
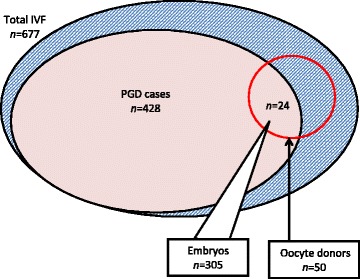
**To determine frequency and source of chromosomal error in “good prognosis” IVF cases, embryos (** ***n*** **= 305) from patients undergoing both anonymous donor oocyte IVF and PGS of embryos were subjected to comprehensive chromosomal screening.**

During the 12 month review period ending December 2013, a total of 676 IVF cases proceeded to oocyte retrieval at this unit. Of these, 50 were anonymous oocyte donors undergoing ovum pick-up. A total of 428 patients requested PGD during the study interval. Intersecting these two patient sub-sets identified 24 IVF cases which included both anonymous oocyte donation and PGS (see Figure 1). Analysis of this group revealed that 305 embryos underwent biopsy and full molecular karyotyping. The mean (±SD) age of recipient females in this study population was 42.5 ± 4.0 (range 35-52) years. Mean (±SD) age was 24.0 ± 2.7 (range 20-29) years for oocyte donors (*n* = 24).

The partners of the intended parents had a mean (±SD) age of 44.3 ± 7.1 (range 25-58 years). Average sperm concentration and motility were 52.8 M/mL (range 2.4-135 M/mL) and 40.8% (range 2-81%), respectively.

In this study group, the mean (±SD) number of oocytes which underwent ICSI was 17.7 ± 7.8 (range = 6-35), and this yielded an average of 15.1 ± 6.7 2*pn* zygotes per patient (range = 6-32). Most embryos (86%) were biopsied on day three, while the remainder (14%) were biopsied on day five. Although the number of blastocyst biopsies was relatively small (*n* = 44), it was possible to record embryo ploidy as a function of biopsy timing. Using this approach, we found the incidence of missed calls (“no signal”) on chromosomes to be significantly higher among embryos biopsied at day three, resulting in reduced reporting efficiency for this group compared to the blastocyst biopsy group (92 vs. 100%; *p* = 0.05).

**Figure 2:**
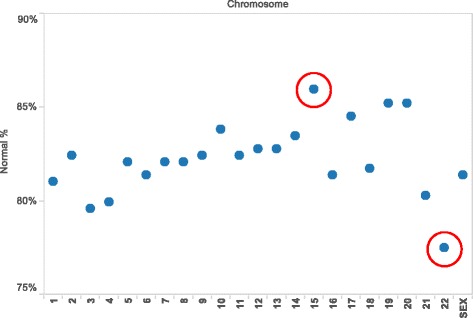
**Distribution of aneuploidy as a function of specific chromosomal error measured in embryos (** ***n*** **= 305) produced from anonymous donor oocyte IVF cycles.** All chromosomes demonstrated some ploidy error in this study, although chromosome 15 and chromosome 22 (red circles) were found to have the highest and lowest rates of abnormality, respectively.

**Figure 3:**
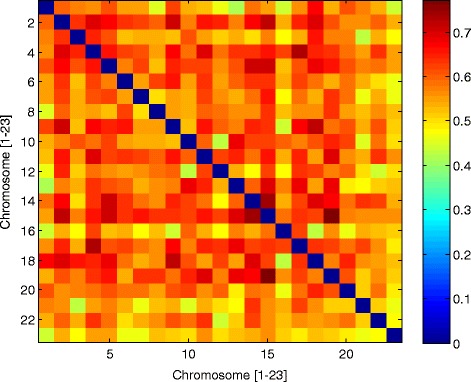
**Pairwise correlations of autosomal aneuploidy by mean square contingency (Phi) coefficient (observed in 305 embryos derived from anonymous donor oocyte IVF treatments) reveal a wide distribution of error among all chromosomes.**

Assessment of all embryos produced from oocytes contributed by an anonymous donor identified euploidy in 133 of 284 (46.8%) of embryos with full chromosomal reporting (*i.e*. zero “no calls”). Complete data on all 23 chromosome pairs was reported for 93.1% of embryos sampled (284 of 305). Considering all embryo chromosomes, mean error rate was 18%. A chromosome-specific analysis found some error in each chromosome, but chromosome 22 was most often affected. In contrast, chromosome 15 was the least likely to have an abnormality in this population (see Figure 2). The relatively high Phi correlation coefficients (see Figure 3) among embryo chromosome pairs with aneuploidy (*r* = 0.60, range 0.42-0.77; *p* < 0.01 by Chi-square test) indicates that chromosomes tend to have multiple and simultaneous errors (complex aneuploidy).

**Figure 4:**
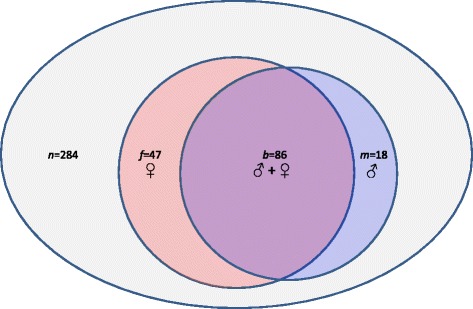
**In this investigation, most embryos contained chromosomal error contributed by both genetic parents.** However, the anonymous oocyte donor was responsible for a greater number of chromosomal abnormality compared to the sperm source. Here, distribution of aneuploidy origin (by gamete source) for embryos produced from anonymous donor oocyte IVF is summarized.

When analysis was confined only to those embryos with no missed calls for any chromosome, errors attributable to a maternal source (*i.e*., from the oocyte donor) were noted in 133 of 284 embryos (46.8%). Conversely, an embryo genetic abnormality of paternal origin was present in 104 of 284 embryos (36.6%). Among all aneuploid embryos (*n* = 151), chromosomal errors from both genetic parents (*i.e*., oocyte donor and sperm source) were present in 57.0% (see Figure 4). While oocyte donor age ranged from 20-29 years, some genetically abnormal embryos were produced from donors of each age and there was no correlation between oocyte donor age and embryo aneuploidy. Likewise, these data did not confirm a correlation between embryo aneuploidy and male partner age or any semen parameter.

## Discussion

The arrival of oocyte donation preceded PGS in clinical practice, and was originally offered as a treatment for premature ovarian failure or oophorectomy [6]. Egg donation is now commonly in use for many settings besides diminished ovarian reserve, including its use to circumvent transmission of severe genetic disorder(s) in the birth mother to her offspring [7]. While the corrosive effect of age on female infertility can be successfully assuaged by use of oocytes donated by a younger (presumably more fertile) woman [8], the benefit of this “genetic rescue” is difficult to calibrate in clinical practice. Of note, the degree of aneuploidy in embryos derived from egg donation was surprisingly high when only a few chromosomes were evaluated [5].

A recent retrospective study covering 12 years of data collected from anonymous oocyte donor applicants found that genetic abnormalities resulted in a significant number of candidates being rendered ineligible to participate in their oocyte donor program [9]. We agree with such screening, and, like many institutions, require any potential anonymous oocyte donor to first undergo a careful genetic testing regime before entering the roster of active oocyte donors. Indeed, all of the anonymous donors who supplied oocytes for the current study already had been screened for hundreds of genetic disorders in advance of their accession into our egg donor group. However, despite this reassuring clearance (and in the absence of any obvious reproductive pathology) the rate of chromosomal error among embryos produced from their eggs was still 55%.

Previous research has attempted to characterize the role of “defective” gametes resulting in generation of abnormal embryos using an egg-sharing model, where one IVF patient agrees to share her eggs with another IVF patient [10]. It is unclear if this treatment strategy is ideal for the poor prognosis IVF patient, since what she ultimately gets is simply bad eggs from another infertility patient. Such a study is unsatisfying experimentally because the variable of oocyte pathology cannot be controlled if all oocytes are generated by other patients with a variety of infertility diagnoses.

Enhanced clarity on this issue was provided when aneuploidy rate for eight chromosomes in embryos derived from young (<35 yrs) oocyte donors using fluorescence in-situ hybridization analysis was studied. Using this approach, all oocytes were provided by healthy women who did not have any infertility diagnosis. The authors reported considerable variation between donor cycles with nearly one-third having <30% genetically normal embryos [5]. Starting from these data where less than half of the embryo’s chromosomes had been evaluated, our work built on this foundation to screen all 23 pairs of embryo chromosomes in an anonymous donor oocyte IVF setting. Importantly, since behavior of each parental allelic group is a function of the underlying chromosome copy number of the embryo, and because these modifications may be satisfactorily estimated from additional allelic content contributed by (or omitted from) either the oocyte donor or the sperm source, we were able to supply additional information on the parental origin of the genetic problems identified in the embryos derived therefrom.

Earlier research has shown a significantly higher observed pregnancy loss rate among IVF patients with age ≥40 compared to women younger than age 40 [11], establishing that the distribution of genetic error in embryos is a function of maternal age. This physiologic process of natural ovarian senescence has been sidestepped for many years by using oocytes provided by younger donors [12]. With further refinement of donor oocyte protocols, acceptance of this treatment in routine IVF practice has increased greatly over the last decade, and when donor oocytes are used the likelihood of an excellent IVF outcome seems independent of recipient age [13]. In the United States, the incidence of twins is markedly higher among anonymous oocyte donor IVF cycles compared to IVF using native (autologous) oocytes (37 vs. 29%, respectively), which provides direct evidence that most clinics are not following a current recommendation by the American Society for Reproductive Medicine which encourages single embryo transfers when oocyte donor age is young [14]. Moreover, there is international consensus that elective single embryo transfers are appropriate for oocyte donor-recipient cycles where the donor has good prognosis and when good quality embryos are available [15].

Until now, comprehensive chromosomal screening has not been applied to embryos of donor oocyte origin to quantify the level of genetic abnormality present in such embryos. If ever the domain of anonymous donor oocyte IVF were regarded as a realm where the frequency of embryo aneuploidy could be dismissed as unimportant, the current study suggests otherwise and highlights an important supporting role for PGS in this setting. But the basic assumption that preimplantation embryo testing adds diagnostic value to any IVF patient or that this reliably improves reproductive outcomes has not been without challenge [16,17]. Particularly when preimplantation genetic assessment evaluates only a limited number of embryo chromosomes, the process has been derided as “…the first ever widely introduced routine IVF practice which actually harmed IVF cycle outcomes” [18].

While more data are needed to settle this debate, our study does contribute some new observations on human embryo genetics. Here, we focused on the specific topic of parental origin with respect to chromosomal errors harbored by IVF embryos. Our observation that a high rate of embryonic genetic anomaly could be traced back to the oocyte donor was not anticipated. Thus, even when the age of the oocyte source is low, the traditional view that most chromosomal errors are of maternal origin caused by malsegregation in the first meiotic division [19] appears to remain valid.

Our report has some limitations which should be acknowledged. Our data come from a retrospective analysis as an initial step to analyze readily accessible existing data. We aimed to produce a hypothesis about aneuploidy rate in embryos derived from anonymous donor oocytes which could then be tested prospectively. Retrospective work has the potential for incomplete documentation, unrecoverable or unrecorded data, and variance in the quality of information recorded. The reliability of data entry is considered high for this sample, and the proportion of incomplete records was marginal. Also, because our sample was limited and represented the chance event of an IVF patient using anonymous donor oocytes also incorporating pre-implantation testing of embryos produced from this treatment, it is uncertain if these findings can generalize to all anonymous donor egg IVF cases. However, a secondary chart review for our study population did not reveal any obvious characteristic which may have influenced the patient’s decision to include PGS in her IVF treatment. Perhaps the high economic cost of IVF in general [20] (and donor oocyte treatment in particular) introduced some “access bias”, as only the most affluent IVF patients might have been able to afford this particular treatment. It would be interesting to query the remaining donor oocyte IVF patients in this series who declined PGS (*n* = 26), to understand better why they decided not to request genetic testing for their embryos; this represents an area of future research here. Finally, our analysis of male factor data was confined to age of the recipient’s husband and only two semen parameters (sperm concentration and motility). We did not include sperm DNA fragmentation data in this study, although this has not yet been correlated with embryo ploidy [21].

## Conclusion

IVF with anonymous oocyte donation remains a highly effective treatment for many patients with low ovarian reserve. While this approach effects a partial retreat of the embryo aneuploidy problem, complete surrender seems impossible—even when oocytes from donors as young as 20 years of age are used. Further prospective studies are needed utilizing comprehensive chromosomal screening of embryos obtained from oocyte donor IVF cycles.

## Methods

### Study design

This retrospective investigation reviewed selected data from all in vitro fertilization (IVF) cases at HRC Fertility (Orange County, Calif.) in 2013 to identify the subset of patients where PGS was performed on embryos derived exclusively from anonymous oocyte donors. IRB approval was sought although the proposal was classified as exempt because the study reviewed data already collected and no specific patient identifiers were recorded. PGS results were collated with parental DNA obtained immediately before IVF (*i.e.,* from the anonymous egg donor and the sperm source) for chromosome-specific assessments. This approach permitted mitotic and meiotic copy errors to be differentiated for each chromosome among all embryos tested, thus providing information on the specific parental source of embryo aneuploidy.

### Oocyte donor and patient (recipient) selection

Anonymous oocyte donors had completed comprehensive medical and psychological evaluation as described previously [22]. Additionally, donors underwent a genetic evaluation and were required to have a normal result (no mutations) on an expanded carrier test [23] before enrollment. Recipients had their initial reproductive endocrinology consultation and monitoring at our facility, and all baseline laboratory tests were within normal limits. Anonymous oocyte donor counseling was provided by an accredited psychologist before starting gonadotropins. Each recipient selected her anonymous oocyte donor via secure internet portal with an electronic lock-out mechanism to prohibit multiple recipients from accessing the aggregate donor pool at the same time. A dedicated nurse coordinator was available to facilitate oocyte donor selection in all cases. Following registration of each provisional donor-recipient match, the corresponding anonymous oocyte donor entry was deleted from the donor library, thus creating a 1:1 ratio for each recipient and their anonymous oocyte donor (*i.e*., no two IVF recipients utilized oocytes from the same anonymous donor for this analysis).

The anonymous oocyte donor commenced controlled ovarian hyperstimulation and transvaginal ultrasound-guided oocyte collection followed 36 h after s.c. hCG administration as previously described [24]. Sperm from the recipient’s partner was used to fertilize all freshly retrieved eggs obtained from the anonymous oocyte donor; intracytoplasmic sperm injection (ICSI) was performed in all cases.

For all records reviewed for this study, recipient and (male) partner age were tabulated, as was age of the anonymous oocyte donor. Sperm concentration and sperm motility were calculated as an average of two semen analyses performed no more than six months before treatment. The following laboratory parameters were also evaluated: number of oocytes fertilized (via ICSI), number of two pronuclear (2*pn*) zygotes produced, number of embryos biopsied, day of biopsy, and number of euploid embryos. In addition, the number and frequency of error observed in each chromosome was recorded, with reference to the (genetic) parental origin of the abnormality, as described previously [25].

### Ovarian stimulation and fertilization

Before commencing gonadotropin therapy, oocyte donors underwent transvaginal ultrasound evaluation with re-measurement of serum FSH, LH and estradiol on d3 of the index cycle. Pituitary downregulation was achieved with GnRH-agonist administered on d21 of the cycle immediately preceding treatment, as previously described [24]. Periodic transvaginal ultrasound and serum estradiol measurements were used to track follicular growth and thickness of endometrial lining. When ≥3 follicles reached 19 mm mean diameter, periovulatory hCG was administered by subcutaneous injection of recombinant hCG (250 μg Ovidrel®, Merck Serono; Geneva, Switzerland) with oocyte retrieval performed under transvaginal ultrasound guidance 35-36 h later. Following removal of all cumulus cells, ICSI was performed and normal fertilization was verified 16-18 h after injection by presence of two pronuclei and two polar bodies.

### Embryo culture and biopsy

For these embryos, biopsy was performed either on the morning of day three or at the blastocyst stage (day five). Biopsy at day three was completed after laser assisted hatching followed by removal of a single blastomere. Extended embryo culture occurred in Global single-step medium (IVF on Line; Guilford, CT) to blastocyst stage. On d3 when embryos were at the 6–8 cell stage, a Lycos laser (Hamilton Thorne; Beverly, MA) was used to create a circular 6-9 μ diameter opening in the zona pellucida. This breach enabled biopsy of trophectoderm (TE) on d5 rapidly. Between 3–5 herniated TE cells were gently aspirated by pipette and, when necessary, freed from the blastocyst by application of laser pulses. Harvested TE cells were washed in PBS and placed within a PCR tube with 2.5 μl 1× PBS.

### Cell isolation, DNA amplification, and genotyping procedures

Genetic material was obtained from oocyte donors (buccal swabs), recipient’s husband (peripheral venipuncture), and embryos (either single-cell day 3 blastomere biopsy or multi-cell day 5 trophectoderm biopsy). Single tissue culture (polymorphonuclear leukocytes) and egg donor buccal cells were isolated using a sterile tip attached to a pipette and stereomicroscope (Leica; Wetzlar, Germany). For fresh day 3 embryo biopsy, individual blastomeres were separated via micromanipulator after zona pellucida hatching by Hamilton-Thorne Lycos laser; a micromanipulator was also used to isolate individual sperm cells. Except for sperm, single cells for analysis were washed × 4 with buffer (PBS buffer, pH 7.2; Life Technologies, Carlsbad CA). Multiple displacement amplification (MDA) with proteinase K buffer (PKB) was used for this procedure; cells were placed in 5 μl PKB (Arcturus PicoPure Lysis Buffer, 100 mM DTT, 187.5 mM KCl, 3.75 mM MgCl_2_, 3.75 mM Tris-HCl) incubated at 56°C × 1 h, followed by heat inactivation at 95°C × 10 min and held at 25°C × 15 min. MDA reactions were incubated at 30°C × 2.5 h and then 65°C × 10 min.

Genomic DNA from buccal tissue was isolated using the QuickExtract DNA Extract Solution (Epicentre; Madison WI). Template controls were included for the amplification method. Amplified single cells and parental tissue were genotyped using the Infinium II (Illumina; San Diego, CA) genome wide single nucleotide polymorphism (SNP) arrays (CytoSNP 12 chip). The standard Infinium II protocol was used for parent samples and Genome Studio (http://res.illumina.com/documents/products/technotes/technote_gentrain2.pdf) was used for allele calling. For single cells, genotyping was accomplished using an Infinium II genotyping protocol.

### Establishing copy number and haplotype phasing

Because some commercial software packages use heterozygosity to determine copy number, and high rates of allele drop-out with preferential amplification in single cell measurements can cause unpredictable heterozygosity (regardless of chromosome copy number), performance is poor when calling copy number on single cell data. Accordingly, previous investigators [25] developed a chromosome copy number classification algorithm in MATLAB (MathWorks; Natick, MA), predicated on parental genotypes and the observed distribution of unprocessed single cell microarray channel intensities collated by parental origin [26,27].

In brief, this approach is based on prior work [25] whereby the statistical behavior of each parental group differs as a function of the underlying chromosome copy number of the embryo. These changes are predictable and derive from additional allelic content that is contributed by (or missing from) each parent [25]. Moreover, rank statistics are examined for each parental context and compared to the expected orderings under the various chromosome copy number possibilities. Next, the probability is examined for each parental context that could have swapped rank by random chance to establish copy number and calculate confidences [25,28].

Detection of three unmatched haplotypes adds additional confidence to a trisomy call, as many chromosome copy number errors are meiotic and will be associated with this configuration. Accordingly, this method included parental information with high-confidence disomic single cell measurements on offspring and recombination probabilities to determine the parental chromosome phase. A maximum likelihood estimator algorithm was used to phase full chromosomes for all parental genotype contexts. Possible haplotypes in single cell measurements are then evaluated to detect meiotic trisomies.

Segmental copy imbalances were detected by dividing each chromosome into five segments, with the aforementioned algorithm applied to each section independently. If any segments differ in copy number with high confidence, then the corresponding chromosome is flagged. Note that the reported copy number for chromosomes with a segmental imbalance is reflective of the call on the majority of the chromosome, even if part of the chromosome shows gain or loss. Thus, depending on size, segmental copy imbalances may reduce composite confidence of the complete chromosome call. However, confidences on chromosomes with segmental imbalances may still be high if the deletion is relatively small and/or the remainder of the chromosome is called with very high confidence [25].

Individual chromosome means and standard deviations of normalized microarray probe intensities were used to call chromosome copy number. For each single cell measurement, a training set of single cell amplification microarray measurements was used to normalize probe intensities across each chromosome. An algorithm was next used to compute the most likely chromosome state for all the single cell amplification microarray data.

### Statistical analysis

Data were aggregated, analyzed, and visualized with Tableau 8.2 (Tableau Software; Seattle, WA). To estimate a reference population's aneuploidy rate and the donor (maternal genetic) aneuploidy contribution, a binomial proportion confidence interval was used on each proportion estimate using the Wald test. When sample size was small (defined as min[np, n(1-p)] <5), an adjusted Wald method was used to improve estimation accuracy [29]. For this analysis, the confidence level was set at 95% by default (90 for aneuploidy rate comparisons). To compare two sample ratios, the 2-proportion z-test was used for large samples (defined as min[np, n(1-p)] > =5); Fisher’s exact test was used when sample size was small.
